# EFFECT OF L-VALINE TREATMENT ON SIRTUIN (SIRT1 AND SIRT2) ISOFORMS

**DOI:** 10.1093/geroni/igac059.2667

**Published:** 2022-12-20

**Authors:** Shakshi Sharma, Xiaomin Zhang, Gohar Azhar, Jeanne Wei

**Affiliations:** University of Arkansas for Medical Sciences (UAMS), Little Rock, Arkansas, United States; University of Arkansas for Medical Sciences (UAMS), Little Rock, Arkansas, United States; University of Arkansas for Medical Sciences (UAMS), Little Rock, Arkansas, United States; University of Arkansas for Medical Sciences (UAMS), Little Rock, Arkansas, United States

## Abstract

L-valine is one of the essential branched-chain amino acids (BCAAs) required for synthesis of proteins in human body. It promotes muscle growth and tissue repair and is important for immune function. Recent data indicate that BCAAs can activate sirtuins expression and elevate mitochondrial biogenesis and fatty acid oxidation in both adipocytes and myotubes thereby increasing life span. Sirtuins are a conserved family of proteins, play a critical role in maintaining metabolic health by deacetylating many target proteins in numerous tissues, and regulate mitochondrial function and the aging process. Due to multiple effect of sirtuins on aging, we sought to determine whether the addition of valine might enhance sirtuin gene expression. We utilized the C2C12 skeletal muscle cell line grown on physiological normal glucose (100mg/dL) media. The cells were treated with two different concentrations of valine (0.5 and 1.0mM) for different time intervals (18 and 24). Gene expression of sirtuin 1 (SIRT1) and sirtuin 2 (SIRT2) isoforms were determined by RT-PCR. The results showed increased expression of the sirtuin gene isoforms after treatment with valine. Relative expression varies with in different isoforms of SIRT1 (v1 and v2) and SIRT2 (v1, v2 and v3). Among all, SIRT1 v1 and SIRT2 v1 showed maximum expression as compared to the other isoforms used in the study. Our study showed that adequate supplementation of L-valine enhanced sirtuin gene expression, which may promote healthy muscles and healthy aging.

